# Psychosocial outcomes following dual-task high-velocity exercise training in older adults: a secondary analysis of an 18-month cluster randomized controlled trial

**DOI:** 10.1093/geronb/gbag140

**Published:** 2026-07-14

**Authors:** Jamie L Tait, Rachel L Duckham, Timo Rantalainen, Catherine M Milte, Luana C Main, Caryl A Nowson, Kerrie M Sanders, Dennis R Taaffe, Keith D Hill, Gavin Abbott, Robin M Daly

**Affiliations:** Institute for Physical Activity and Nutrition (IPAN), School of Exercise and Nutrition Sciences, Deakin University, Geelong, Victoria, Australia; Australian Institute for Musculoskeletal Science (AIMSS), Department of Medicine-Western Health, The University of Melbourne, Melbourne, Victoria, Australia; Department of Medicine, The Royal Melbourne Hospital, The University of Melbourne, Melbourne, Victoria, Australia; Finnish Institute of High Performance Sport KIHU, Jyväskylä, Finland; Institute for Physical Activity and Nutrition (IPAN), School of Exercise and Nutrition Sciences, Deakin University, Geelong, Victoria, Australia; Institute for Physical Activity and Nutrition (IPAN), School of Exercise and Nutrition Sciences, Deakin University, Geelong, Victoria, Australia; Institute for Physical Activity and Nutrition (IPAN), School of Exercise and Nutrition Sciences, Deakin University, Geelong, Victoria, Australia; Department of Medicine, Western Health, The University of Melbourne, Melbourne, Victoria, Australia; Exercise Medicine Research Institute and School of Medical and Health Sciences, Edith Cowan University, Joondalup, Western Australia, Australia; Rehabilitation Ageing and Independent Living (RAIL) Research Centre, Monash University, Melbourne, Victoria, Australia; Institute for Physical Activity and Nutrition (IPAN), School of Exercise and Nutrition Sciences, Deakin University, Geelong, Victoria, Australia; Institute for Physical Activity and Nutrition (IPAN), School of Exercise and Nutrition Sciences, Deakin University, Geelong, Victoria, Australia

**Keywords:** Dual-task training, Health-related quality of life, Physical activity, Life satisfaction

## Abstract

**Objectives:**

Power and resistance training can improve older adults’ perceived health, and while cognitive training has mixed benefits, combined effects remain uncertain. This secondary analysis of an 18-month cluster randomized controlled trial investigated whether dual-task functional power training (DT-FPT) improves psychosocial outcomes in older adults at risk for falls.

**Methods:**

Twenty-two independent-living retirement communities (300 residents, ≥65 years) were randomized to 12 months of group-based DT-FPT (6 months supervised + 6 months maintenance; 2/week, 45–60 min) performed with cognitive and/or motor tasks or control (CON), followed by a 6-month follow-up. Health-related quality of life (HR-QoL; SF-36v2), life satisfaction (Personal Wellbeing Index), and psychological distress (Depression Anxiety and Stress Scale [DASS-21]) were assessed at baseline, 6, 12, and 18 months, generating subscale, summary, and composite scores.

**Results:**

Overall, 227 (76%) participants provided data at the 18-month follow-up; mean exercise adherence was 50% at 6 months and 40% at 12 months. At 6 months, DT-FPT showed net benefits compared to CON for physical functioning (1.77 points [95% CI: 0.03, 3.51]; *p = *.047), role-physical (2.71 [0.35, 5.08]; *p = *.025), and role-emotional (2.25 [0.09, 4.40]; *p = *.041) HR-QoL domains. No changes were observed in life satisfaction or psychological distress, but CON showed lower Anxiety DASS-21 subscale scores than DT-FPT at 12 months (−2.0 [−3.22, −0.76]; *p = *.001) and 18 months (−0.93 [−1.84, −0.02]; *p = *.045).

**Discussion:**

DT-FPT delivered to older, retirement-living adults at risk of falls may improve select HR-QoL domains, potentially reducing perceived physical limitations in daily activities, but shows no sustained effects or impact on life satisfaction.

Ageing often involves lifestyle changes, including retirement, downsizing, and relocation, as well as a deterioration in physical function, which can adversely affect quality of life (QoL) (Arent et al., 2000). In addition, age-related cognitive decline is associated with reduced social interaction and lower QoL (Maki et al., 2014). In terms of physical function, age-related losses in muscle mass, strength, and functional performance contribute to frailty, fall risk, and reduced independence, all of which negatively impact health-related quality of life (HR-QoL) (Rizzoli et al., 2013). Given that HR-QoL reflects one’s perceived health across physical, mental, and social domains, interventions targeting both cognitive and physical function may offer significant benefits for HR-QoL in older adults.

Regular exercise training improves mental health, stress, and QoL in older adults (Arent et al., 2000; Netz et al., 2005), though specific modes such as aerobic training show mixed effects on HR-QoL (Awick et al., 2015; Kelley et al., 2009). Meta-analyses indicate that progressive resistance training (PRT) significantly enhances HR-QoL in older adults (Effect Size: 0.31–0.81), improving perceptions of mental health, bodily pain, physical functioning, general health, and social functioning (Hart & Buck, 2019; Khodadad Kashi et al., 2023). However, its effects on other domains, such as vitality, role limitations due to physical and emotional problems, and physical and mental composites, remain inconsistent, likely due to variations in intervention characteristics (e.g., duration, intensity, frequency of training). Power training, a high-velocity form of PRT, improves muscle power (i.e., the ability to generate force rapidly, which is distinct from peak muscle strength/force and muscle mass) as well as physical and perceived function (Katula et al., 2008; Ramirez-Campillo et al., 2016). Given that muscle power may be more strongly associated with independence and QoL than muscle mass or strength alone (Katula et al., 2008), power training may be more beneficial than traditional PRT for maintenance of independence. However, the optimal exercise approach for improving diverse HR-QoL domains remains unclear (Awick et al., 2015; Kelley et al., 2009), underscoring the need for interventions targeting both physical and cognitive health.

Dual-task exercise, integrating simultaneous cognitive and motor tasks, demonstrates potential for improving HR-QoL in older adults (Dunsky, 2019; Dunsky et al., 2017; Trombini-Souza et al., 2022), though benefits vary by the training mode used (e.g., functional strength and balance training, coordination training, step training, or combinations of these approaches). Dual task programs spanning 8–24 weeks, incorporating step-training, balance and coordination performed alongside auditory cues and cognitive tasks (e.g., mathematical challenges) have produced improvements in domains such as physical functioning (Trombini-Souza et al., 2022) and general health (Dunsky et al., 2017) compared to a control group that was required to variably shift attention between cognitive and motor tasks, and a non-exercise control group, respectively. In contrast, 8–12-week dual-task multimodal training interventions, integrating physical and cognitive tasks such as resistance, aerobic and balance exercises with memory or arithmetic challenges and motor tasks (Jardim et al., 2021; Rezola-Pardo et al., 2019), have shown limited within-group gains in global HR-QoL (Rezola-Pardo et al., 2019), physical functioning, vitality, general health, and role limitations due to physical problems (Jardim et al., 2021). Dual-task functional balance training, which enhances neuromuscular control (i.e., integration of sensory and motor systems for movement regulation) and improves static and dynamic stability, has been linked to improvements in HR-QoL (Dunsky, 2019). Moreover, functional balance and power training more closely mirror daily activities (e.g., rising from a chair, climbing stairs, stepping over obstacles), enhancing transferability and potentially supporting more consistent improvements in HR-QoL. Therefore, a longer-duration, multimodal dual-task functional power and balance training program may offer greater benefits for older adults’ HR-QoL, but this remains unexamined. Furthermore, the potential benefits of dual-task functional balance training may be particularly relevant for older adults at risk of falls, who commonly experience deficits in neuromuscular control, dynamic stability, and dual-task performance. Despite this, no HR-QoL studies have examined dual-task training in cohorts explicitly identified as being at risk of falls. The only studies with tangentially related inclusion criteria recruited participants with good balance (Trombini-Souza et al., 2022) or relatively high functional independence (Rezola-Pardo et al., 2019), limiting the relevance of current findings to those at risk of falls.

Satisfaction with Life (SWL), a cognitive assessment of well-being, is positively associated with physical activity in older adults (Rejeski & Mihalko, 2001), though structured exercise interventions yield mixed results (Awick et al., 2015; Rejeski & Mihalko, 2001). Multimodal training (Brown et al., 2009; Kohut et al., 2006), high-velocity PRT (Katula et al., 2008), and cognitively engaging activities like dancing (Kosmat & Vranic, 2016) show promise as strategies to improve SWL, while cognitive training alone appears ineffective (Shatil et al., 2014). Emerging dual-task methods, including square stepping and visual cue-based walking (Sok et al., 2021) may enhance SWL and reduce depression, yet the effects of dual-task functional power and balance training remain unexplored. Therefore, this secondary analysis of an 18-month cluster randomized controlled trial (RCT) aimed to examine whether a 12-month dual-task functional power training (DT-FPT) program could improve psychosocial well-being (HR-QoL, SWL, and psychological distress [symptoms of depression, anxiety, and stress]) compared to usual care in older adults at risk of falls living independently in retirement living communities.

## Materials and methods

### Study design

This secondary analysis draws on an 18‑month cluster RCT conducted in 22 independent‑living retirement communities involving older adults at elevated risk of falls, and examines outcomes in HR-QoL, SWL and psychological distress. As reported previously (Tait et al., 2024), villages were randomized to either a multicomponent dual‑task functional power‑training program (DT‑FPT; 11 villages, 156 participants) or a usual‑care control group (CON; 11 villages, 144 participants). The primary aim of the trial was to evaluate intervention effects on falls across three sequential 6‑month phases (supervised training, maintenance, and follow‑up) (Daly et al., 2015). Recruitment occurred over 14 months in three cohorts (February 2014–April 2015) (Duckham et al., 2018), with baseline and follow‑up assessments completed within each village. Cohort sizes were 82 participants across eight villages, 104 participants across six villages, and 114 participants across eight villages. Cluster randomization was performed at the village level to avoid contamination, using block allocation (blocks of two) stratified by village size (<75 or ≥75 residents), using computer-generated numbers generated in Microsoft Excel by an independent researcher. Testing personnel were blinded to group allocation, except for one researcher responsible for participant communication (J.L.T.). Ethical approval was granted by Deakin University Human Research Ethics Committee (EC 2013‑051), and the trial was registered with the Australian and New Zealand Clinical Trials Registry (ACTRN12613001161718).

### Participants

A total of 300 older adults (81 men and 219 women) were recruited from 22 retirement living communities (villages) across metropolitan Melbourne and regional Victoria, Australia. Recruitment and screening procedures have been detailed elsewhere (Daly et al., 2015; Duckham et al., 2018; Tait et al., 2024). Briefly, individuals were eligible if they were aged ≥65 years, scored ≥3 on a falls-risk algorithm (adapted from Sanders et al. [2009]), made no more than two errors on the Short Portable Mental Status Questionnaire, were proficient in English, and were able to walk at least 50 meters with minimal or no assistance. Exclusion criteria were: undertaking ≥150 min/week of moderate-to-vigorous physical activity or more than one weekly structured resistance or balance session in the previous 3 months; musculoskeletal, neurological, acute, terminal, or unstable cardiovascular/respiratory conditions; a limb fracture within the past 3 months; or uncorrected visual impairment. All eligible participants completed the Exercise and Sports Science Australia exercise-screening tool, with medical clearance required for any “Yes” responses before commencing the intervention. Written informed consent was obtained prior to participation.

### Intervention

Villages allocated to the exercise program completed two supervised group sessions each week, each lasting 45–60 min with 8–10 participants per group, delivered on non-consecutive days for 6 months. Sessions were conducted on-site by exercise professionals who had completed a standardized train-the-trainer program to ensure fidelity of delivery. Detailed methods are published elsewhere (Daly et al., 2015; Tait et al., 2024). In brief, each session comprised four components: (1) a warm-up incorporating rhythmic and range-of-motion movements (e.g., marching); (2) 2–3 balance and mobility tasks designed to replicate everyday functional demands (e.g., multidirectional weight shifts, obstacle-course walking); (3) high-velocity FPT involving 5–6 lower-limb exercises (e.g., squats, lunges), at least one upper-limb exercise (e.g., bent-over rows), and core/postural work (e.g., fit-ball sit-ups); and (4) a cool-down. Progression for stepping (2 × 10–20 repetitions, up to 50) and FPT (2 × 10–15 repetitions) targeted an intensity of 4–6 (moderate to hard) on the 10-point Borg Rating of Perceived Exertion scale. Dual-task elements were incorporated by combining balance/mobility and FPT exercises with concurrent cognitive (e.g., anagrams), visual (e.g., memory recall), or motor tasks (e.g., weighted-ball throws). The 6-month program commenced with a 2-week familiarization period followed by three 8-week training blocks (Daly et al., 2015).

### Step-down maintenance program and follow-up

Following 6 months of supervised training, the DT-FPT group entered a 6-month “step down” maintenance training phase with less supervised support (Daly et al., 2015). One weekly supervised class was delivered by a trainer funded by the research team, with participants encouraged to add an independent brisk walk each week. Villages could opt for a second trainer-led session (village/resident-funded); only one village did so, and three conducted weekly self-directed sessions. Following this phase, all participants were followed for 6 months without further support from the research team.

### Control group

Control group participants (CON) continued their usual activities and received standard care, which comprised ongoing medical management and participation in community-based activities as directed by their treating healthcare professionals. They did not receive the structured exercise program and instead received a falls-prevention booklet (Department of Health: “Don’t fall for it–falls can be prevented”) and were encouraged to meet physical activity guidelines (150 min of moderate to vigorous physical activity), supported by pamphlets and initial contact with researchers.

### Health-related quality of life (SF-36® v2™)

The Short Form 36 version 2 (SF-36v2) was used to measure HR-QoL (Ware & Sherbourne, 1992) and reported using Australian norm-based scores according to previously published guidelines (Hawthorne et al., 2007). The SF-36v2 consists of 36 items that contribute to eight domains of HR-QoL: physical functioning, role limitations resulting from physical health problems, bodily pain, social functioning, general mental health, role limitations resulting from emotional problems, vitality, and general health problems (Ware & Sherbourne, 1992). Summary scores for each of the eight domains and two overall summary scores (physical component summary [PCS] and the mental component summary [MCS]) were used for analysis. A global composite of HR-QoL was also created by averaging the weighted means of the eight domains. Analysis of SF-36v2 data utilized published “weightings” most relevant to this population (Hawthorne et al., 2007). Higher scores indicate fewer limitations.

### Satisfaction with life (Personal Wellbeing Index, PWI)

SWL was assessed via the Personal Wellbeing Index (PWI) (Cummins et al., 2003). The PWI assesses SWL as a whole using a single-item measure that asks “How satisfied are you with your life as a whole?” (0 = *Very Dissatisfied*; 5 = *Neutral*; 10 = *Very Satisfied*), multiplied by 10 for analysis. It also includes eight domain-specific items reflecting aspects of QoL: standard of living, achievement in life, personal health, personal relationships, personal safety, community-connectedness, future security, and spirituality/religion. A composite life satisfaction score was derived by averaging the eight domain-specific ratings and multiplying by 10.

### Depression Anxiety and Stress Scale (DASS-21)

The Depression Anxiety and Stress Scale (DASS)-21 is a 21-item self-report tool assessing depression, anxiety, and stress symptoms over the past week (Henry & Crawford, 2005). Subscale scores range from 0 to 42, with higher scores indicating greater symptom severity.

### Anthropometry

Anthropometric measures were collected at baseline to characterize the sample. Height was assessed to the nearest 0.1 cm using a portable stadiometer (Surgical and Medical PE87), and body weight to the nearest 0.01 kg with TANITA scales (BC-418, Tanita Co., Japan). Body mass index (BMI) was derived as weight (kg) divided by height (m) squared.

### Health and medical history, and medication use

Participant characteristics were obtained using a study-specific questionnaire that captured medical history (from a predefined list of 33 conditions), current and past medication use, ethnicity (derived from birthplace and parental ethnic background), education, and smoking behavior. Cardiometabolic risk was classified as present when participants met at least one of the following criteria: diagnosed hypertension or antihypertensive medication use, lipid-lowering medication use, type 2 diabetes, or a previous diagnosis of myocardial infarction, heart disease, or angina.

### Adverse events

Adverse events were defined as any unintended health-related signs, symptoms, or illnesses that emerged or worsened during the trial. Trainers documented events occurring during or after exercise sessions (e.g., illness or injury) and classified them as “definitely related,” “possibly related” or “not related” to the exercise intervention. An unblinded researcher additionally interviewed participants in both groups at 3, 6, 12, and 18 months to identify and verify adverse events associated with DT-FPT or the trial beyond usual care.

### Exercise adherence

Exercise adherence was calculated as the percentage of prescribed sessions completed, based on attendance at supervised sessions during the initial 6-month intervention (two sessions per week) and the step-down phase (one session per week), using completed exercise cards.

### Statistical analyses

The sample size for this study was based on the primary aim of the trial to reduce the rate of falls (Daly et al., 2015). After accounting for cluster randomization, the required sample size was 280 participants recruited from approximately 15 villages. Due to higher-than-expected attrition in Cohort 1, the planned sample size was increased to 300, which provided over 80% power (2-tailed, *p* < .05) to detect modest effect sizes (e.g., Cohen’s *d* = 0.3) in the secondary HR-QoL outcomes, based on previous intervention effects (Cassilhas et al., 2007; de Vreede et al., 2007). Primary analyses were conducted on an intention-to-treat basis, with missing data handled using complete case analysis, assuming data were missing completely at random. Data were checked for normality prior to the main analyses. Intervention effects on HR-QoL, SWL, and DASS-21 were evaluated using linear mixed models with robust standard errors. Group allocation (DT-FPT or CON) was included as a fixed effect, while random intercepts were specified for participants nested within retirement villages to account for clustering. The unit of analysis was the individual participant. Intervention effects on HR-QoL, SWL, and psychological distress scores were initially assessed by adjusting for baseline values (Model 1), followed by additional adjustment for age, sex, education, and cardiometabolic status (Model 2). Within-group changes were examined using random-intercepts linear mixed-effects models conducted for each group, with change from baseline as the outcome. Per-protocol analyses were also undertaken. Although ≥80% adherence to DT-FPT was prespecified, only 36 participants (23%) met this threshold. The criterion was therefore modified to ≥50% adherence during the initial 6-month program (equivalent to one session per week) or ≥50% adherence to the step-down period. Thus, these findings are considered exploratory.

Additional sensitivity analyses were conducted using multiple imputation to handle missing data, valid under the assumption of data missing at random. Missing baseline and follow-up values were imputed via chained equations, generating 50 datasets to ensure stable estimates (Lee & Carlin, 2010). Auxiliary variables, including sex, age, education, and cardiometabolic status, were incorporated into the imputation models to improve imputation accuracy.

All analyses were performed using STATA version 17.0 (StataCorp, College Station, TX, USA). Descriptive statistics are presented as mean ± standard deviation, median (IQR), or frequency (%). Statistical significance was defined as *p *< .05. No adjustments were applied for multiple testing or co‑primary outcomes, to minimize the risk of over‑correction. Intervention effects are reported as mean estimates with 95% CIs to enable assessment of their relative importance.

## Results

### Participant characteristics

As reported previously (Tait et al., 2024), 300 men and women aged 65 to 96 years (mean ± *SD*; 77.4 ± 6.8) were recruited across 22 retirement living communities. One DT-FPT participant withdrew after baseline due to health-related issues and requested removal of their data, leaving 299 participants in the final sample (DT-FPT, *n = *155; CON, *n = *144). Clusters (villages) averaged 14 participants (range 5–23). Sex distribution differed between groups at baseline (DT-FPT: 65% women; CON: 82% women; Table 1), reflecting natural variation due to village-level clustering, and thus was included as a covariate for all outcome measures. At baseline, 287 participants (96%) had ≥1 chronic condition, 119 (40%) were classified as obese (BMI >30; Table 1), and 43% were ex- or current smokers (10 current).

**Table 1:** Baseline characteristics of participants randomized to the dual-task functional power training (DT-FPT) and the usual care control (CON) groups.

Characteristics	DT-FPT (*n = *155)	CON (*n = *144)
**Number of clusters (retirement communities)**	11	11
**Number of participants per cluster**	14 (9, 17)	11 (7, 16)
**Women, *n* (%)**	101 (65%)	118 (82%)
**Age, years**	77.2 ± 6.6	77.7 ± 7.2
**Height, cm**	163.2 ± 9.1	160.0 ± 8.0
**Weight, kg**	77.7 ± 16.0	74.3 ± 14.1
**Body mass index (kg/m^2^)**	29.1 ± 5.2	29.0 ± 4.9
**Smoking status, *n* (%)**		
Current/Ex-Smoker	72 (46%)	56 (39%)
Non-smoker	82 (53%)	86 (60%)
**Presence of chronic health conditions^a^**	150 (97%)	137 (96%)
Number of conditions in those with a condition	3 (2, 4)	3 (2, 4)
Presence of cardiometabolic conditions	144 (94%)	130 (92%)
Presence of neurological conditions	18 (12%)	20 (14%)
Presence of motor-related impairment	94 (61%)	92 (65%)
**Caucasian, *n* (%)**	151 (98%)	139 (98%)
**Education, *n* (%)**		
Primary/Some High School	57 (37%)	57 (40%)
Completed High School/Technical Trade Certificate	50 (32%)	49 (34%)
University/Tertiary level	47 (30%)	36 (25%)

*Note*. CON = control; DT-FPT = dual-task functional power training. Values are mean ± standard deviation or n (%), except for participants per cluster and number of health conditions, which are presented as median (25th, 75th percentile).

a Missing data: DT-FPT: *n = *1 (0.6%), CON: *n = *2 (1.4%). Cardiometabolic conditions included hypertension, heart disease, hypercholesterolemia, type 1 and type 2 diabetes, chronic kidney failure, kidney disease or stones, hypo- or hyperthyroidism, asthma, past or current cancer, and vitamin D abnormality; neurological conditions included stroke, Parkinson’s disease, epilepsy, and multiple sclerosis; and motor-related impairments included arthritis, rheumatoid arthritis, and osteoarthritis.

Lipid-lowering and antihypertensive medications were used by 49% and 71% of participants, respectively (Table S1). Over the study period, no statistically significant between-group differences were observed for changes to medication usage (type, dose, or number), number of diseases (total or new), or disease prevalence. For individual HR-QoL domains, all mean norm-based scores were below 50, with the exception of the mental health domain. Relative to normative data for Australian adults aged over 75 years (Hawthorne et al., 2007), the participants were on average within ±0.4 *SD* for all measures (Table 2). For PWI at baseline, participants on average scored at the higher end of the range (mean ± *SD*: 79.8 ± 13.1) relative to the current normative range for Australians over 65 years (77.5—79.7 points) (Capic et al., 2016). Despite limited normative data, participants generally scored lower on the DASS-21 subscales than a normative Australian sample (*n = *497, mean age 42.1), with scores for depression (3.7 ± 5.4 vs 5.1 ± 7.7), anxiety (3.8 ± 5.0 vs 3.4 ± 5.6), and stress (5.4 ± 6.0 vs 8.0 ± 8.4) (Crawford et al., 2011). However, these comparisons should be interpreted cautiously given the age differences between samples and the limited availability of age-matched normative data.

**Table 2:** Mean baseline HR-QoL norm-based and PWI scores, within-group changes relative to baseline, and net between-group differences after 26 weeks: dual-task functional power training (DT-FPT) vs usual care control (CON) groups.

Variables	DT-FPT		CON		Intervention effects		
	*n*	Mean ± *SD* or (95% CI)	*n*	Mean ± *SD* or (95% CI)	Estimated group differences (95% CI) ^a^	*p*	
						M1	M2
**Physical functioning**							
Baseline	154	40.3 ± 11.1	143	39.5 ± 11.4			
Δ6 months	118	0.29 (−0.78, 1.36)	118	−**1.63 (**−**2.98,** −0.27)*	**1.92 (0.30, 3.55)**	**.020**	**.047**
Δ12 months	113	0.44 (−1.23, 2.12)	114	−1.04 (−2.47, 0.39)	1.54 (−0.62, 3.70)	.162	.245
Δ18 months	113	−**1.44 (**−**2.62,** −0.25)*	114	−**2.29 (**−**3.53,** −**1.04)**‡	0.94 (−0.70, 2.57)	.263	.418
**Role physical**							
Baseline	154	45.0 ± 10.1	143	44.2 ± 10.5			
Δ6 months	118	0.55 (−0.46, 1.56)	118	−1.73 (−3.62, 0.17)	**2.40 (0.21, 4.59)**	**.032**	**.025**
Δ12 months	113	−0.95 (−2.66, 0.77)	114	−0.81 (−2.13, 0.52)	−0.07 (−2.53, 2.40)	.959	.917
Δ18 months	113	−**2.53 (**−**4.27,** −**0.79)**†	114	−**2.42 (**−**3.68,** −**1.17)**‡	−0.02 (−1.99, 1.95)	.982	.794
**Bodily pain**							
Baseline	154	45.0 ± 11.0	143	43.4 ± 11.9			
Δ6 months	118	0.19 (−1.87, 2.25)	118	−0.70 (−2.30, 0.90)	1.09 (−1.65, 3.84)	.436	.421
Δ12 months	113	−0.88 (−3.16, 1.41)	114	−0.38 (−2.43, 1.66)	−0.36 (−3.45, 2.73)	.820	.643
Δ18 months	113	−0.74 (−2.65, 1.16)	114	−0.61 (−2.24, 1.03)	0.20 (−2.47, 2.88)	.882	.944
**General health**							
Baseline	154	47.6 ± 8.1	142	46.8 ± 9.1			
Δ6 months	118	0.38 (−0.50, 1.26)	118	0.74 (−0.33, 1.81)	−0.26 (−1.51, 0.99)	.684	.913
Δ12 months	113	0.23 (−1.00, 1.45)	114	−0.03 (−1.17, 1.11)	0.39 (−1.22, 2.00)	.633	.373
Δ18 months	113	−0.37 (−1.88, 1.14)	114	0.13 (−1.29, 1.55)	−0.33 (−2.28, 1.61)	.738	.856
**Vitality**							
Baseline	154	48.8 ± 9.7	143	48.9 ± 9.6			
Δ6 months	118	−0.04 (−1.62, 1.54)	118	0.18 (−1.49, 1.86)	−0.36 (−2.40, 1.68)	.730	.767
Δ12 months	113	−0.90 (−1.95, 0.14)	114	−0.93 (−2.41, 0.54)	0.05 (−1.74, 1.84)	.955	.788
Δ18 months	113	−1.28 (−2.33, −0.22)*	114	−0.56 (−1.86, 0.73)	−0.88 (−2.56, 0.80)	.304	.253
**Social functioning**							
Baseline	154	49.2 ± 9.5	143	49.3 ± 9.3			
Δ6 months	118	−0.09 (−1.27, 1.08)	118	−**2.28 (**−**4.07,** −0.49)*	1.96 (−0.16, 4.08)	.070	.142
Δ12 months	113	−1.10 (−3.34, 1.13)	114	−**1.08 (**−**2.02,** −0.14)*	−0.23 (−2.35, 1.89)	.835	.696
Δ18 months	113	−**3.03 (**−**5.58,** −0.48)*	114	−**2.16 (**−**3.70,** −**0.62)**†	−0.94 (−3.45, 1.58)	.466	.296
**Role emotional**							
Baseline	154	46.4 ± 12.1	143	46.9 ± 11.5			
Δ6 months	118	1.29 (−0.54, 3.13)	118	−1.33 (−3.07, 0.41)	**2.49 (0.37, 4.62)**	**.021**	**.041**
Δ12 months	113	−1.01 (−3.23, 1.21)	114	−1.83 (−3.84, 0.19)	0.71 (−2.12, 3.54)	.623	.517
Δ18 months	113	−2.62 (−5.58, 0.34)	114	−**3.17 (**−**6.10,** −0.24)*	0.59 (−2.98, 4.16)	.747	.984
**Mental health**							
Baseline	154	49.6 ± 9.0	143	50.0 ± 9.0			
Δ6 months	118	0.60 (−0.44, 1.63)	118	−0.20 (−1.71, 1.32)	0.27 (−1.29, 1.84)	.731	.890
Δ12 months	113	0.31 (−1.14, 1.77)	114	−0.28 (−1.54, 1.07)	0.07 (−1.92, 2.07)	.941	.787
Δ18 months	113	−0.62 (−2.59, 1.34)	114	−0.77 (−2.38, 0.84)	−0.32 (−2.67, 2.02)	.787	.419
**Physical component summary**							
Baseline	154	43.0 ± 10.4	142	41.7 ± 10.4			
Δ6 months	118	0.07 (−0.84, 0.98)	118	−1.00 (−2.26, 0.26)	1.20 (−0.31, 2.72)	.119	.071
Δ12 months	113	−0.33 (−1.95, 1.29)	114	−0.50 (−1.61, 0.61)	0.38 (−1.67, 2.44)	.714	.686
Δ18 months	113	−**1.28 (**−**2.51,** −0.04)*	114	−**1.18 (**−**2.29,** −0.07)*	0.08 (−1.57, 1.74)	.920	.721
**Mental component summary**							
Baseline	154	51.0 ± 9.4	142	52.1 ± 9.3			
Δ6 months	118	0.69 (−0.41, 1.79)	118	−0.62 (−2.01, 0.77)	0.90 (−0.33, 2.14)	.152	.214
Δ12 months	113	−0.63 (−2.15, 0.90)	114	−1.05 (−2.50, 0.40)	0.07 (−1.87, 2.01)	.940	.945
Δ18 months	113	−1.80 (−4.03, 0.43)	114	−1.72 (−3.69, 0.24)	−0.48 (−3.05, 2.08)	.711	.435
**Global HR-QoL**							
Baseline	154	46.5 ± 7.7	143	46.1 ± 7.8			
Δ6 months	118	0.39 (−0.33, 1.10)	118	−0.91 (−2.11, 0.30)	1.26 (−0.07, 2.59)	.064	.069
Δ12 months	113	−0.48 (−1.44, 0.47)	114	−0.79 (−1.66, 0.07)	0.30 (−1.00, 1.60)	.650	.659
Δ18 months	113	−**1.58 (**−**2.91,** −0.25)*	114	−**1.52 (**−**2.35,** −**0.68)**‡	−0.04 (−1.51, 1.42)	.954	.787
**SWL**							
Baseline	153	78.6 ± 16.7	143	79.0 ± 18.0			
Δ6 months	117	0.94 (−1.23, 3.11)	118	1.19 (−0.50, 2.87)	−0.90 (−3.96, 2.16)	.564	.419
Δ12 months	112	−0.89 (−2.73, 0.95)	114	1.23 (−1.43, 3.89)	−2.24 (−5.86, 1.39)	.226	.130
Δ18 months	112	0.63 (−2.13, 3.38)	114	0.00 (−2.22, 2.22)	0.23 (−3.74, 4.20)	.911	.957
**PWI composite**							
Baseline	154	80.3 ± 12.2	143	79.3 ± 14.1			
Δ6 months	118	0.63 (−0.84, 2.09)	118	0.33 (−0.88, 1.54)	0.28 (−1.57, 2.12)	.768	.622
Δ12 months	113	−0.54 (−2.23, 1.14)	114	1.32 (−0.55, 3.18)	−1.76 (−4.30, 0.77)	.173	.064
Δ18 months	113	−0.03 (−1.42, 1.35)	114	0.26 (−1.52, 2.04)	−0.20 (−2.54, 2.14)	.868	.776

Note. CI = confidence interval; CON = control; DT-FPT = dual-task functional power training; HR-QoL = Health-related quality of life; PWI = Personal Wellbeing Index; SD = standard deviation; SWL = Satisfaction with Life. Satisfaction with Life was a single-item measure multiplied by 10. The PWI composite is the average of PWI subdomains. For HR-QoL and life satisfaction outcomes, higher scores indicate better outcomes. For change scores, positive values reflect improvement while negative values reflect decline. Baseline values are reported as means ± *SD*. Within-group and estimated between-group differences are presented as means (95% CI), adjusted for clustering. *p*-values for group differences were derived from linear mixed models with random intercepts for villages: M1 (Model 1; adjusted for baseline values and clustering) and M2 (Model 2; adjusted for age, sex, education, cardiometabolic status, baseline values, and clustering). Bolded values indicate statistically significant within-group changes relative to baseline after adjusting for clustering (*p < .05 vs baseline; ^†^*p *< .01 vs baseline; ^‡^*p *≤ .001 vs baseline), and statistically significant estimated between-group differences.

a Values represent coefficients from Model 1, rather than by subtracting within-group changes from baseline for CON from within-group changes for DT-FPT at each time point.

### Study attrition and missing data

**Figure 1:**
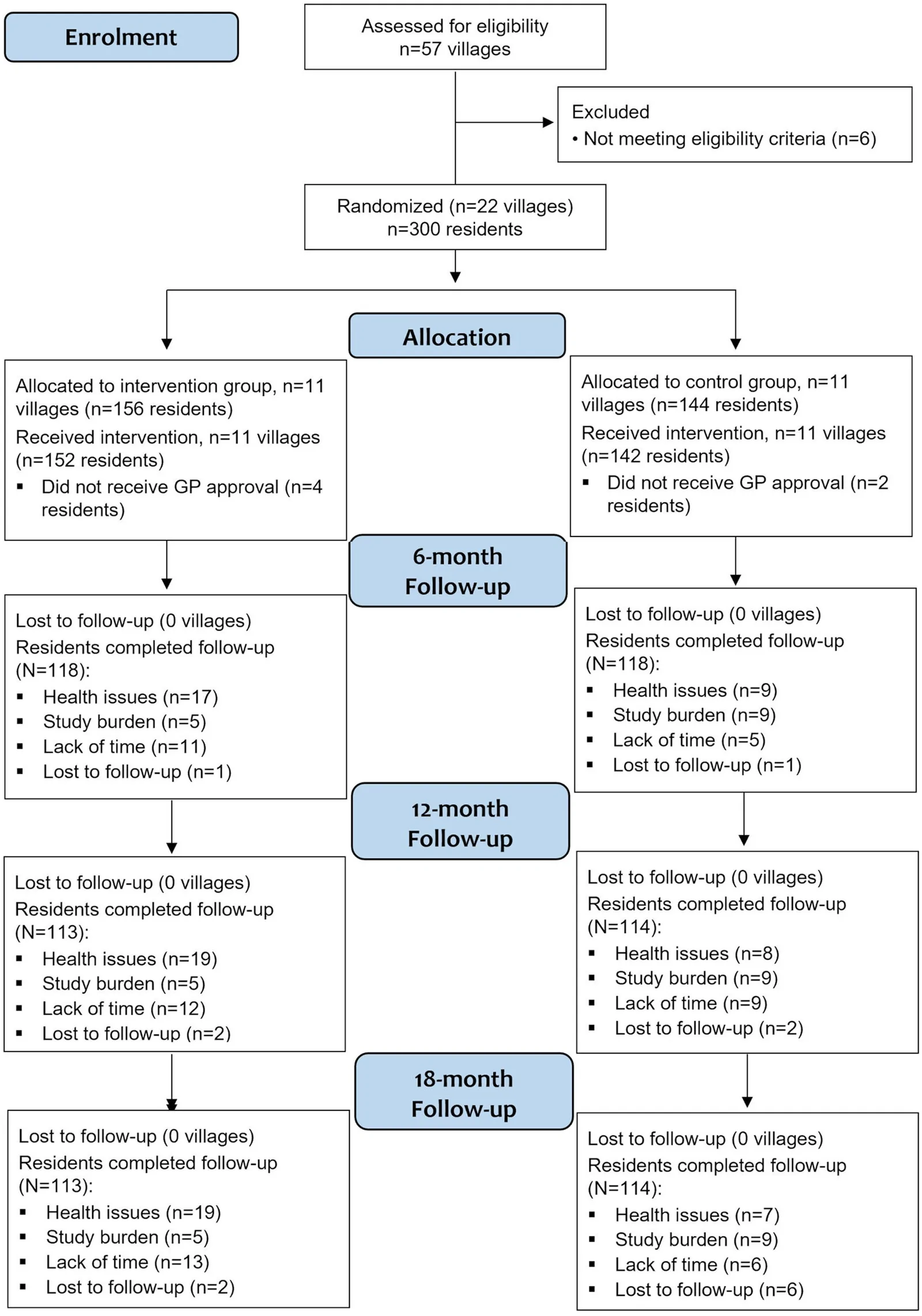
Study flow diagram of villages and participants over the 18-month study period.

Reasons for withdrawal or lack of follow-up at each time point are presented in Figure 1.

In total, 64 (21%) participants (DT-FPT: 24%; CON: 18%) did not provide questionnaire data at 6-month testing. Of these, 49 participants withdrew from the trial (DT-FPT: *n = *31 [10% of the total sample]; CON: *n = *18 [6%]). A further 15 participants did not provide data due to ill health (*n = *12) or were unable to be contacted/lost to follow-up (*n = *3). At 12 months, 56 had withdrawn (DT-FPT: *n = *35 [12%]; CON: *n = *21 [7%]), while 17 did not provide data due to health issues (*n = *6), lack of time (*n = *6), or loss to follow-up (*n = *5). At 18 months, 60 had withdrawn (DT-FPT: *n = *38 [13%]; CON: *n = *22 [7%]), while 13 did not provide data due to health (*n = *2), lack of time (*n = *2), or loss to follow-up (*n = *9). Hence, the final numbers for analysis at 6, 12, and 18 months were 236, 227, and 227, respectively.

One participant from the DT-FPT group withdrew after baseline and requested data removal, resulting in a final sample of 299 participants (DT-FPT: *n = *155; CON: *n = *144). Missing baseline data for the presence of chronic health conditions occurred in one DT-FPT participant (0.6%) and two CON participants (1.4%). At baseline, two participants did not complete the SF-36v2, PWI, or DASS-21 questionnaires (DT-FPT: *n = *1, 0.6%; CON: *n = *1, 0.7%). One participant in CON provided incomplete SF-36v2 responses and was excluded from analyses involving SF-36v2 summary scores (0.7%), and one DT-FPT participant did not complete the optional SWL item from the PWI at any time point (0.6%). Overall, baseline missing data were low (<2% across measures) and distributed fairly evenly across groups, while missing follow-up data due to non-attendance at assessment sessions was approximately 23%–25% from baseline to 6 months and remained stable thereafter.

### Exercise adherence and adverse events

As previously reported (Tait et al., 2024), mean adherence to the initial 6-month exercise program was 50% ± 32% (median = 58%, range: 0%–98%)—about one session per week—and 12 of 155 (7.7%) DT-FPT participants reported 14 musculoskeletal or health complaints. These included joint pain (knee [*n = *1], shoulder [*n = *2], back [*n = *3], foot [*n = *1]), skin sensitivity from ankle weights (*n = *2), dizziness (*n = *3), bronchitis that was exacerbated by training (*n = *1), and muscle strain (*n = *1). Four events were deemed related to the program, seven possibly related, and three unrelated, with most resolving or requiring only minor management. Of the 14 events, exercise intensity was reduced in 11 cases, the program was temporarily discontinued in two cases (weeks 17–18 and 13–14), and no action was required in one case. Mean adherence during the 6-month step-down period was 40% ± 36% (median 43%, range 0%–100%), equivalent to less than one session per week. When excluding participants who withdrew by 6 months (*n = *31), adherence among those remaining (*n = *125) was 50% ± 33% (median 60%, range 0%–100%) based on attendance at supervised sessions. Mild adverse events were reported during the step-down maintenance period, including knee discomfort (*n = *1) and transient light-headedness (*n = *1); these were considered possibly related to the intervention and required a reduction in exercise intensity. No participants withdrew from the program due to injury, and all who experienced an adverse event completed subsequent follow-up testing.

### Health-related quality of life and satisfaction with life

**Figure 2:**
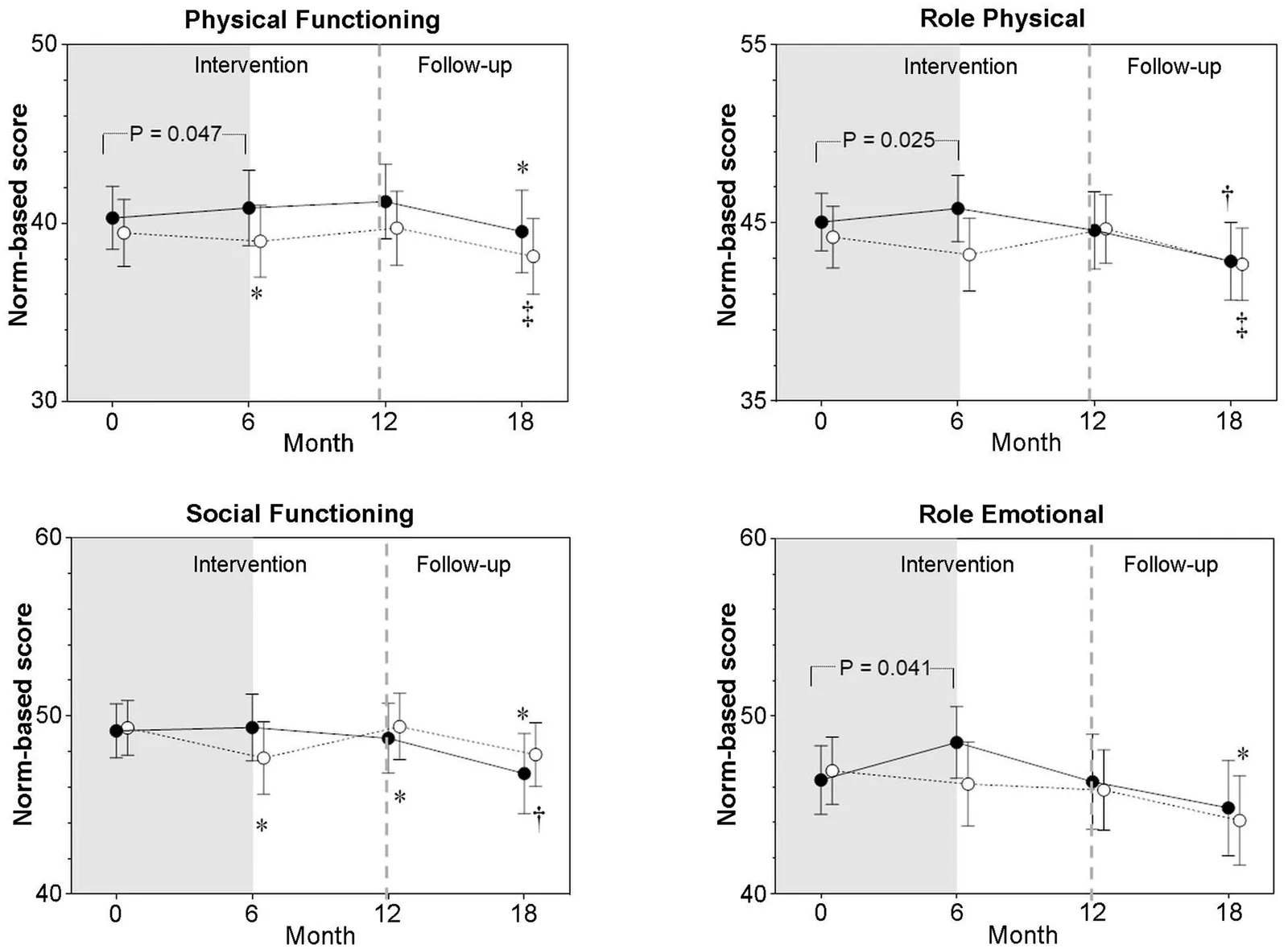
Mean norm-based scores for HR-QoL domains at baseline, 6- and 12-month intervention time points, and after the follow-up period (18 months) in the dual-task functional power training (DT-FPT) and usual care control (CON) groups. *Note*. CON = control; DT-FPT = dual-task functional power training; HR-QoL = health-related quality of life. Closed dots = DT-FPT; open dots = CON. Scores >50 indicate better-than-average HR-QoL or functioning. Error bars indicate 95% confidence intervals. Within-group change relative to baseline, with significant estimated between-group differences after adjusting for age, sex, education, cardiometabolic status, baseline values, and clustering, denoted by line and *p*-value (Model 2; *p < .05; †*p* < .01; ‡*p* ≤ .001). Grey shaded area represents the 6-month supervised intervention period; the subsequent 6-month period represents the step-down intervention phase; dashed line indicates the end of the intervention at 12 months.

Baseline means and adjusted within- and between-group SF-36v2 outcomes are presented in Table 2 and Figure 2, with raw scores in Table S2. In the fully-adjusted model (Model 2), after 6 months, DT-FPT participants experienced an estimated mean net benefit for the domains of physical functioning (1.77 points [95% CI: 0.03, 3.51]; *p = *.047), role-physical (2.71 points [95% CI: 0.35, 5.08]; *p = *.025), role-emotional (2.25 points [95% CI: 0.09, 4.40]; *p = *.041), and a trend for benefits for global HR-QoL (*p* = .069). These domains reflect reduced health-related limitations in physical function and daily work, and fewer limitations in daily activities due to emotional problems. There were no net differences between groups in any SF-36v2 domain at 12 or 18 months. At 12 months, there was a trend for a benefit for CON compared to DT-FPT for the PWI composite score (−2.36 points [95% CI: −4.86, 0.14]; *p = *.064); no other differences were detected for SWL.

For the 6-month per-protocol analyses (≥50% compliance after 6 months), results were consistent after adjusting for covariates (Model 2), with additional benefits for social functioning (2.83 points [95% CI: 0.05, 5.62]; *p = *.046), PCS (1.95 points [95% CI: 0.34, 3.56]; *p = *.018) and global HR-QoL (1.69 points [95% CI: 0.16, 3.23]; *p = *.030) in favor of DT-FPT compared to CON (Table S3). For per-protocol analyses during the step-down period, the only 12-month difference favored CON on the PWI composite score (2.92 points [95% CI: 0.13, 5.71]; *p = *.040). In sensitivity analyses using multiple imputation, net between-group differences in favor of DT-FPT were only detected for role-physical (2.56 points, [95% CI: 0.23, 4.89]; *p = *.031) at 6 months. Previously reported between-group differences in favor of DT-FPT for physical functioning and role-emotional were no longer statistically significant after adjustment for baseline values, age, sex, education, and cardiometabolic status (Table S4).

#### DASS-21

After adjusting for covariates (Model 2), CON showed significantly greater reductions in anxiety than DT-FPT at 12 months (−2.00 points, [95% CI: −3.22, −0.76]; *p* = 0.001) and 18 months (−0.93 points [−1.84, −0.02]; *p* = .045; Table S5). No other between-group differences were observed. In sensitivity analyses using multiple imputation, there were between-group differences in favor of CON for anxiety at 12 months only (−1.72 points, [95% CI: −2.89, −0.55]; *p* = .004), and reductions in stress at 6 months (−1.17 points, [95% CI: −2.29, −0.05]; *p* = .041) and 12 months (−1.53 points, [95% CI: −2.85, −0.22]; *p* = .022), compared to DT-FPT.

## Discussion

This secondary analysis of an 18-month cluster RCT found that 6 months of supervised DT-FPT improved HR-QoL domains of physical functioning, role-emotional, and role-physical in older adults living independently in retirement communities. These results suggest that this form of training may have the potential to reduce the impact of physical limitations on the daily activities of older adults carrying out their usual role. However, it is important to recognize the lack of broader effects (e.g., general health, vitality, social functioning, or composite scores), as well as the absence of sustained effects at 12 months. This likely reflects the modest adherence to the intervention, the inclusion of a relatively healthy cohort of older adults, and the specific focus of the dual-task exercise program on optimizing physical function.

Physical functionality is a key determinant of QoL in older adults (Rizzoli et al., 2013). DT-FPT improved physical functioning, role-physical and role-emotional scores after the 6-month supervised training phase, but did not alter PCS or other physical health domains (vitality, general health, bodily pain). This suggests DT-FPT may lessen perceived physical and emotional limitations in performing daily activities. These findings likely reflect multitask exercises requiring concurrent attention across movement components (e.g., functional balance, stepping, power training) and visual, cognitive, and motor tasks. Previous dual-task programs integrating step-training, balance and coordination with auditory cues and cognitive tasks (e.g., mathematical challenges) typically report improvements only in physical functioning and general health over 8–24 weeks (Dunsky et al., 2017; Trombini-Souza et al., 2022), likely due to enhanced neuromuscular control and stability, which can improve QoL (Dunsky, 2019). Similarly, functional (e.g., carrying objects, rising from a chair) (de Vreede et al., 2007) and balance training (El-Khoury et al., 2015) alone can improve physical functioning but not role-physical or role-emotional domains in older adults. This supports the principle of training specificity, whereby improvements may be most evident in outcomes closely aligned with the demands of the exercise intervention. In contrast, multimodal interventions combining strength and balance training with cognitive tasks (Jardim et al., 2021; Rezola-Pardo et al., 2019) show inconsistent benefits for physical functioning, vitality, general health, and role limitations in community and residential settings. The inclusion of power training may have supported perceived physical function, consistent with prior research (Katula et al., 2008; Ramirez-Campillo et al., 2016), and may reflect actual gains in functionality and power. Power training, which emphasizes explosive, functional movements, may better reflect the demands of daily activity than traditional strength or aerobic training, and may support the maintenance of functional capacity and perceived physical function in older adults. When combined with balance training, power training may contribute to reductions in perceived physical limitations and improvements in functional independence.

The cognitively demanding components of our dual-task program, such as walking in time to music, attention-demanding cognitive tasks, and multidirectional stepping, may have contributed to improvements in role-physical and role-emotional domains, aligning with prior reviews of cognitively demanding physical interventions (e.g., dance-based exercise, exergaming) (Cacciata et al., 2019; Lu et al., 2024). Our findings indicate that embedding cognitively and physically demanding elements into functional training can improve specific HR-QoL domains. Functional tasks inherently require cognitive engagement, and functional stepping may promote neural efficiency by reducing activation in brain regions such as the prefrontal cortex and hippocampus (Nishiguchi et al., 2015). In addition, dual-task training engages brain regions involved in both cognitive and emotional processing, including the anterior cingulate cortex, which supports emotional regulation and the integration of sensory, motor, and cognitive information (Bush et al., 2000). These neural adaptations may strengthen emotional regulation, which may in turn increase awareness of physical improvements while reducing emotional distress and perceived physical limitations (Skevington & Wright, 2001). Together, these effects may contribute to improvements in the role-emotional and role-physical domains, facilitating greater emotional regulation and reducing the impact of physical limitations on daily functioning among older adults.

Importantly, DT-FPT was largely ineffective at improving several HR-QoL domains and composites (e.g., physical and mental component summaries, global HR-QoL), likely due to the modest adherence, relatively low training intensity, and a relatively healthy cohort. Despite tailored, supervised, group-based exercise designed to optimize adherence, attendance remained modest (∼50% during the supervised intervention phase across all 155 participants, and 60% among the 125 DT-FPT participants retained at 6-month follow-up), which likely limited broader intervention effects. Per-protocol analyses of participants with ≥50% adherence (mean 75%) revealed additional gains in social functioning, PCS, and global HR-QoL compared to controls. Previous studies in retirement communities report similar adherence (∼39%–51%) (Lord et al., 2003; Merom et al., 2016), with one reporting no HR-QoL gains after 12 months of twice-weekly social dancing (Merom et al., 2016). In our study, participants averaged one 45–60-min session per week (∼20–26 hrs over 6 months). Conversely, interventions involving cognitively challenging dance, high-intensity training PRT or power training and ≥2 sessions per week (75%–91% adherence) over 12–24 weeks (Cassilhas et al., 2007; Hui et al., 2009; Ramirez-Campillo et al., 2016), have shown broader HR-QoL improvements, suggesting a minimum of two sessions or ∼180 min per week may be required to improve multiple HR-QoL domains (Kaushal et al., 2019).

The modest HR-QoL effects of DT-FPT may reflect its low-moderate intensity in addition to frequency. Meta-analyses indicate moderate-intensity PRT or aerobic training more reliably improves HR-QoL domains such as mental health, vitality, bodily pain and the MCS composite (Hart & Buck, 2019; Khodadad Kashi et al., 2023; Netz et al., 2005), likely via increased serotonin release, stress hormone regulation and enhanced self-efficacy (Barbour et al., 2007). This may explain the lack of mental health and MCS improvements, consistent with null findings from similar dual-task (Jardim et al., 2021; Trombini-Souza et al., 2022), functional (de Vreede et al., 2007), and balance (El-Khoury et al., 2015) programs in older adults. Limited improvements in specific HR-QoL domains also likely reflect the cohort’s baseline scores, near or above Australian norms for adults ≥75 years (Hawthorne et al., 2007). These limited changes align with evidence that individuals with lower baseline functioning or greater chronic disease burden experience more pronounced benefits (Kaushal et al., 2019; Khodadad Kashi et al., 2023). Similarly, clinically meaningful changes (≥0.5 *SD* or ≥5 units), typical in chronic disease populations (Norman et al., 2003), were not observed in our sample, underscoring baseline health and function as key moderators of HR-QoL responsiveness.

No changes in life satisfaction were detected in our study, likely due to both high baseline levels and the stability of this construct over time, even following major life events (Fujita & Diener, 2005). Our cohort’s scores exceeded Australian norms for older adults (79.8 vs 77.5 for adults aged >66 and 79.7 for those 76 and older) (Capic et al., 2016), possibly reflecting the secure, socially supportive environment of retirement living communities, which may have produced ceiling effects that limited detection of intervention-related gains (Awick et al., 2015). Social interaction in exercise settings supports psychological well-being, but existing social programs in participating retirement villages may have already elevated life satisfaction, reducing scope for further improvement. The stability of life satisfaction also likely explains the absence of changes in our study, aligning with prior exercise trials reporting null effects using varied life satisfaction measures and prescriptions (e.g., aerobic, functional, PRT) (Awick et al., 2015; Katula et al., 2008; Solberg et al., 2013). Familiar, socially oriented activities like lawn bowls or cards may enhance life satisfaction more than exercise or cognitive training (Shatil et al., 2014), while intervention effectiveness may depend on outcomes older adults value, such as functional performance or self-efficacy, which influence both life satisfaction and HR-QoL (White et al., 2009). Future research should explore how social environments in retirement communities support physical activity and assess both HR-QoL and life satisfaction in dual-task trials.

Several factors may explain the unexpected reductions in anxiety symptoms in the control group at 12 and 18 months. Regression to the mean and potential for differential attrition are plausible contributors: baseline anxiety was higher in controls, and those who withdrew from the control group by 12 months had higher baseline anxiety than both DT-FPT withdrawers and retained controls (4.8 vs 3.4 and 4.2, respectively). This may have biased the observed change. Additionally, reducing supervised sessions from 2 to 1 per week between 6 and 12 months may have been perceived as reduced support, fostering unmet expectations and transiently increasing anxiety in DT-FPT, while control participants may have benefited from usual‑care routines or attention effects. It is also possible that participation in the exercise intervention introduced transient perceptions of performance-related pressure or expectations to improve (Eynon et al., 2019), which were not present in the control condition. However, performance-related anxiety was not directly assessed in this study, and therefore this interpretation remains speculative. These factors warrant cautious interpretation and further investigation.

This study has several strengths, including being the first RCT to examine HR-QoL and SWL outcomes following a 12-month dual-task functional power-training program in older adults at falls risk. Cluster randomization limited intervention contamination, and researcher blinding minimized bias. The larger sample size, powered for the trial’s broader aims (falls), is a notable strength. This study also has limitations. First, adherence to the DT-FPT program was modest (40%–50%), but consistent with other exercise studies in independently living older adults (Lord et al., 2003; Merom et al., 2016), which may have reduced intervention effects. The cognitive and motor complexity of the dual-task program may have contributed to lower engagement and enjoyment, particularly among individuals with functional or emotional limitations. Notably, participants who completed the study had higher baseline HR-QoL and life satisfaction than those who withdrew, suggesting selective loss of individuals with greater potential for improvement, which may have influenced outcomes. Incorporating participant feedback into dual-task program design may help improve engagement and retention in future studies. Second, the sample was relatively homogeneous, relatively healthy, well-educated, and 98% Caucasian, limiting generalizability to more diverse, frail, or cognitively impaired populations. While underlying health conditions may have influenced outcomes, exploratory analyses suggested that arthritis modified intervention effects at 6 months, with participants with arthritis in the DT-FPT group showing significantly greater improvements across several psychosocial domains (e.g., role- physical, general health, social functioning, physical and mental component summaries, and overall HR-QoL). However, these analyses were not pre-specified and should be interpreted with caution. Overall, the influence of underlying health status on intervention effectiveness, including potential enhanced responsiveness among individuals with health conditions, warrants further investigation. Third, although the PWI demonstrates good reliability and correlates with the SWL Scale, its sensitivity to detect change in exercise-based interventions requires further evaluation. In addition, only self-perceived physical function outcomes were reported and not objective performance measures. Finally, the absence of adjustments for multiple comparisons may increase the risk of type I error.

In conclusion, this 18-month cluster RCT in older adults at increased falls risk incorporating a 12-month intervention with 6-month follow-up underscores the potential of DT-FPT to improve select HR-QoL domains, including physical functioning, role-emotional, and role-physical. However, no sustained effects were observed over a subsequent 6-month step-down maintenance phase and follow-up, and no significant changes were observed in life satisfaction or psychological distress. These findings suggest dual-task functional exercise training may help mitigate physical limitations affecting daily activities, but the lack of broader HR-QoL benefits highlights the need for further research into long-term effects, adherence strategies, and the role of social and environmental factors in shaping intervention outcomes.

## Supplementary Material

gbag140_Supplementary_Data

## Data Availability

The datasets, analytic procedures, and materials utilized in this research are not available for public access. Furthermore, the studies described in this manuscript were registered on the Australian and New Zealand Clinical Trials Registry (ACTRN12613001161718).
