# Supplementary material for: Psychosocial outcomes following dual-task high-velocity exercise training in older adults: a secondary analysis of an 18-month cluster randomized controlled trial
Source: J Gerontol B Psychol Sci Soc Sci. 2026 Jul 14;81(9):gbag140. doi: 10.1093/geronb/gbag140 (PMC13537523; doi:10.1093/geronb/gbag140)
Supplement: Supplementary file 1 [file gbag140_supplementary_data.docx]

***The Journals of Gerontology, Series B: Psychological Sciences and Social Sciences* Supplementary Material: Tait et al. Psychosocial outcomes following dual-task high-velocity exercise training in older adults: A secondary analysis of an 18-month cluster randomised controlled trial.**

Supplementary Table 1: Medications taken by participants randomised to the dual-task functional power training group (DT-FPT) and control (CON) group at baseline.

| **Medications taken*** | **DT-FPT** | **CON** |
| --- | --- | --- |
| *Antihypertensive, n (%)* | 112 (72%) | 101 (70%) |
| *Lipid-lowering, n (%)* | 82 (53%) | 63 (44%) |
| *NSAIDs, n (%)* | 18 (12%) | 22 (15%) |
| *Anti-depressant, n (%)* | 37 (24%) | 31 (22%) |
| *Diabetic, n (%)* | 16 (10%) | 18 (13%) |
| *Neurological, n (%)* | 4 (3%) | 4 (3%) |
| *Analgesics (non-NSAIDs), n (%)* | 17 (11%) | 16 (11%) |
| *Sleep aids, n (%)* | 11 (7%) | 14 (10%) |

*Note.* * Missing data for DT-FPT: n=1 (0.6%), CON: n=2 (1.4%). NSAID: Nonsteroidal anti-inflammatory drug. Neurological medication included anti-Parkinson and anti-epileptic medication.

Supplementary Table 2: Mean baseline raw HR-QoL values for participants randomised to the dual-task functional power training group (DT-FPT) and control (CON) group.

| **SF-36 domains** | **DT-FPT** | **CON** |
| --- | --- | --- |
| n | 154 | 143 |
| Physical Functioning | 63.4 ± 24.2 | 61.6 ± 24.8 |
| Role Physical | 72.0 ± 25.5 | 69.8 ± 26.4 |
| Bodily Pain | 65.8 ± 23.3 | 62.5 ± 25.4 |
| General Health ^1^ | 66.6 ± 17.6 | 64.8 ± 20.0 |
| Vitality | 58.6 ± 20.1 | 58.9 ± 19.9 |
| Social Functioning | 84.3 ± 21.3 | 84.7 ± 20.8 |
| Role Emotional | 85.3 ± 21.2 | 86.2 ± 20.1 |
| Mental Health | 79.9 ± 15.3 | 80.6 ± 15.4 |

*Note*. Baseline values are means ± SD and represent raw scores from SF-36 scales. ^1^ n=142 for CON group. For HR‑QoL outcomes, higher scores indicate better outcomes.

**Supplementary Table 3:** Mean baseline HR-QoL norm-based and PWI scores and adjusted within-group changes relative to baseline and net between-group differences over the 18-month intervention period in the dual-task functional power training (DT-FPT) and control (CON) groups according to per protocol analyses for those with ≥50% compliance during the 6-month supervised intervention phase.

|  | **DT-FPT** | | **CON** | | **Intervention effects** | | |
| --- | --- | --- | --- | --- | --- | --- | --- |
|  | **n** | **Mean ± SD or**  **(95% CI)** | **n** | **Mean ± SD or**  **(95% CI)** | **Estimated group**  **Differences**  **(95% CI)^a^** | ***p*-values**  **Model 1 \| Model 2** | |
| **Physical functioning** | | |  |  |  |  | |
| Baseline | 91 | 41.7 ± 11.1 | 143 | 39.5 ± 11.4 |  |  | |
| ∆ 6 months | 88 | -0.10 (-1.37, 1.16) | 118 | **-1.63 (-2.98, -0.27)*** | 1.69 (-0.05, 3.43) | 0.057 \| **0.040** |  |
| ∆ 12 months | 85 | 0.30 (-1.30, 1.89) | 114 | -1.04 (-2.47, 0.39) | 1.58 (-0.39, 3.56) | 0.116 \| 0.143 |  |
| ∆ 18 months | 85 | **-1.64 (-3.19, -0.10)*** | 114 | **-2.29 (-3.53, -1.04)**‡ | 0.85 (-1.06, 2.77) | 0.382 \| 0.457 |  |
| **Role physical** | |  |  |  |  |  |  |
| Baseline | 91 | 46.8 ± 9.6 | 143 | 44.2 ± 10.5 |  |  |  |
| ∆ 6 months | 88 | 0.71 (-0.44, 1.85) | 118 | -1.73 (-3.62, 0.17) | 3.13 (0.91, 5.36) | **0.006** \| **0.003** |  |
| ∆ 12 months | 85 | -0.56 (-2.01, 0.90) | 114 | -0.81 (-2.13, 0.52) | 0.68 (-1.31, 2.68) | 0.501 \| 0.351 |  |
| ∆ 18 months | 85 | **-2.76 (-5.00, -0.51)*** | 114 | **-2.42 (-3.68, -1.17)**‡ | 0.18 (-2.07, 2.43) | 0.874 \| 0.666 |  |
| **Bodily pain** |  |  |  |  |  |  |  |
| Baseline | 91 | 45.6 ± 11.0 | 143 | 43.4 ± 11.9 |  |  |  |
| ∆ 6 months | 88 | 0.53 (-2.09, 3.15) | 118 | -0.70 (-2.30, 0.90) | 1.78 (-1.19, 4.75) | 0.240 \| 0.270 |  |
| ∆ 12 months | 85 | -0.83 (-3.73, 2.07) | 114 | -0.38 (-2.43, 1.66) | 0.002 (-3.31, 3.32) | 0.999 \| 0.874 |  |
| ∆ 18 months | 85 | -0.27 (-2.07, 1.52) | 114 | -0.61 (-2.24, 1.03) | 0.95 (-1.67, 3.56) | 0.477 \| 0.365 |  |
| **General health** | |  |  |  |  |  |  |
| Baseline | 91 | 47.8 ± 8.3 | 142 | 46.8 ± 9.1 |  |  |  |
| ∆ 6 months | 88 | 0.77 (-0.36, 1.91) | 118 | 0.74 (-0.33, 1.81) | 0.16 (-1.26, 1.57) | 0.827 \| 0.388 |  |
| ∆ 12 months | 85 | 0.69 (-0.94, 2.33) | 114 | -0.03 (-1.17, 1.11) | 0.86 (-1.01, 2.73) | 0.366 \| 0.177 |  |
| ∆ 18 months | 85 | -0.23 (-1.71, 1.24) | 114 | 0.13 (-1.29, 1.55) | -0.19 (-2.11, 1.73) | 0.844 \| 0.948 |  |
| **Vitality** |  |  |  |  |  |  |  |
| Baseline | 91 | 49.3 ± 9.4 | 143 | 48.9 ± 9.6 |  |  |  |
| ∆ 6 months | 88 | 0.28 (-1.45, 2.02) | 118 | 0.18 (-1.49, 1.86) | 0.04 (-2.17, 2.20) | 0.972 \| 0.931 |  |
| ∆ 12 months | 85 | -0.64 (-1.86, 0.59) | 114 | -0.93 (-2.41, 0.54) | 0.29 (-1.65, 2.23) | 0.768 \| 0.541 |  |
| ∆ 18 months | 85 | -1.10 (-2.40, 0.21) | 114 | -0.56 (-1.86, 0.73) | -0.59 (-2.47, 1.29) | 0.538 \| 0.357 |  |
| **Social functioning** | | |  |  |  |  |  |
| Baseline | 91 | 50.3 ± 9.5 | 143 | 49.3 ± 9.3 |  |  |  |
| ∆ 6 months | 88 | 0.06 (-1.80, 1.93) | 118 | **-2.28 (-4.07, -0.49)*** | **2.54 (0.39, 4.69)** | **0.020** \| **0.046** |  |
| ∆ 12 months | 85 | -0.97 (-3.37, 1.43) | 114 | **-1.08 (-2.02, -0.14)*** | 0.08 (-2.14, 2.30) | 0.942 \| 0.995 |  |
| ∆ 18 months | 85 | -2.19 (-5.03, 0.65) | 114 | **-2.16 (-3.70, -0.62)**† | 0.22 (-2.47, 2.91) | 0.873 \| 0.992 |  |
| **Role emotional** | |  |  |  |  |  |  |
| Baseline | 91 | 48.3 ± 12.1 | 143 | 46.9 ± 11.5 |  |  |  |
| ∆ 6 months | 88 | 0.81 (-1.15, 2.77) | 118 | -1.33 (-3.07, 0.41) | **2.42 (0.30, 4.54)** | **0.026** \| **0.039** |  |
| ∆ 12 months | 85 | -0.28 (-2.57, 2.00) | 114 | -1.83 (-3.84, 0.19) | 1.63 (-0.89, 4.14) | 0.205 \| 0.082 |  |
| ∆ 18 months | 85 | -2.41 (-5.37, 0.55) | 114 | **-3.17 (-6.10, -0.24)*** | 1.12 (-2.40, 4.63) | 0.534 \| 0.647 |  |
| **Mental health** | |  |  |  |  |  |  |
| Baseline | 91 | 49.7 ± 9.0 | 143 | 50.0 ± 9.0 |  |  |  |
| ∆ 6 months | 88 | 0.64 (-0.40, 1.68) | 118 | -0.20 (-1.71, 1.32) | 0.38 (-1.29, 1.99) | 0.642 \| 0.926 |  |
| ∆ 12 months | 85 | 0.30 (-1.63, 2.24) | 114 | -0.28 (-1.54, 1.07) | -0.01 (-2.29, 2.28) | 0.997 \| 0.686 |  |
| ∆ 18 months | 85 | 0.38 (-0.87, 1.63) | 114 | -0.77 (-2.38, 0.84) | 0.74 (-1.27, 2.76) | 0.471 \| 0.974 |  |
| **Physical Component Summary** | | |  |  |  |  |  |
| Baseline | 91 | 44.1 ± 10.6 | 142 | 41.7 ± 10.4 |  |  |  |
| ∆ 6 months | 88 | 0.24 (-0.95, 1.44) | 118 | -1.00 (-2.26, 0.26) | **1.61 (-0.09, 3.31)** | 0.064 \| **0.018** |  |
| ∆ 12 months | 85 | -0.25 (-1.93, 1.43) | 114 | -0.50 (-1.61, 0.61) | 0.63 (-1.39, 2.66) | 0.541 \| 0.430 |  |
| ∆ 18 months | 85 | **-1.49 (-2.93, -0.05)*** | 114 | **-1.18 (-2.29, -0.07)*** | 0.03 (-1.82, 1.87) | 0.977 \| 0.749 |  |
| **Mental Component Summary** | | |  |  |  |  |  |
| Baseline | 91 | 51.7 ± 9.4 | 142 | 52.1 ± 9.3 |  |  |  |
| ∆ 6 months | 88 | 0.62 (-0.51, 1.76) | 118 | -0.62 (-2.01, 0.77) | 0.92 (-0.32, 2.15) | 0.146 \| 0.197 |  |
| ∆ 12 months | 85 | -0.23 (-2.36, 1.89) | 114 | -1.05 (-2.50, 0.40) | 0.39 (-1.83, 2.60) | 0.732 \| 0.710 |  |
| ∆ 18 months | 85 | -0.95 (-2.71, 0.81) | 114 | -1.72 (-3.69, 0.24) | 0.36 (-2.01, 2.73) | 0.766 \| 0.935 |  |
| **Global HR-QoL** | |  |  |  |  |  |  |
| Baseline | 91 | 47.4 ± 7.8 | 143 | 46.1 ± 7.8 |  |  |  |
| ∆ 6 months | 88 | 0.44 (-0.38, 1.27) | 118 | -0.91 (-2.11, 0.30) | **1.44 (0.07, 2.83)** | **0.040 \| 0.030** |  |
| ∆ 12 months | 85 | -0.22 (-1.40, 0.95) | 114 | -0.79 (-1.66, 0.07) | 0.60 (0.81, 2.02) | 0.404 \| 0.292 |  |
| ∆ 18 months | 85 | -1.27 (-2.65, 0.11) | 114 | **-1.52 (-2.35, -0.68)**‡ | 0.35 (-1.19, 1.90) | 0.654 \| 0.644 |  |
| **Satisfaction with life** | | |  |  |  |  |  |
| Baseline | 90 | 79.2 ± 16.6 | 143 | 79.0 ± 18.0 |  |  |  |
| ∆ 6 months | 87 | 0.34 (-2.33, 3.02) | 118 | 1.19 (-0.50, 2.87) | -1.17 (-4.05, 1.71) | 0.426 \| 0.178 |  |
| ∆ 12 months | 84 | -1.19 (-3.82, 1.44) | 114 | 1.23 (-1.43, 3.89) | -2.49 (-6.52, 1.53) | 0.225 \| 0.121 |  |
| ∆ 18 months | 84 | 0.48 (-2.79, 3.74) | 114 | 0.00 (-2.22, 2.22) | 0.44 (-3.73, 4.61) | 0.836 \| 0.863 |  |
| **Personal Well-being Index Composite** | | |  |  |  |  |  |
| Baseline | 91 | 81.5 ± 11.5 | 143 | 79.3 ± 14.1 |  |  |  |
| ∆ 6 months | 88 | 1.13 (-1.08, 3.34) | 118 | 0.33 (-0.88, 1.54) | 0.89 (-1.46, 3.24) | 0.457 \| 0.348 |  |
| ∆ 12 months | 85 | -0.50 (-2.56, 1.56) | 114 | 1.32 (-0.55, 3.18) | -1.69 (-4.54, 1.15) | 0.243 \| 0.085 |  |
| ∆ 18 months | 85 | 0.94 (-0.57, 2.45) | 114 | 0.26 (-1.52, 2.04) | 0.79 (-1.69, 3.28) | 0.532 \| 0.520 |  |

*Note.* Baseline values are reported as means ± SD. For HR‑QoL and life satisfaction outcomes, higher scores indicate better outcomes. For change scores, positive values reflect improvement while negative values reflect decline. Within-group and estimated between-group differences are presented as means with 95% CI, adjusted for clustering. P-values for group differences were derived from linear mixed models with random intercepts for villages: Model 1 (adjusted for baseline values and clustering) and Model 2 (adjusted for age, sex, education, cardiometabolic status, baseline values, and clustering). HR-QoL: Health-related quality of life. The Personal Wellbeing Index (PWI) composite is the average of PWI subdomains. Satisfaction with Life was a single-item measure multiplied by 10. At baseline, SF-36, PWI and DASS-21 questionnaires were not completed by one participant in CON, while one participant provided incomplete responses for some domains of the SF-36 (CON: n=1) and was excluded from analysis for general health, physical component summary and mental component summary composites and the global composite HR-QoL measure. One participant (DT-FPT: n=1) did not wish to answer the optional SWL single-item question at all time points. Bolded values indicate statistically significant within-group changes relative to baseline after adjusting for clustering, and statistically significant estimated between-group differences. ^a^ Estimated between-group differences (95% CIs) represent coefficients from Model 1, rather than by subtracting within-group changes from baseline for CON from within-group changes for DT-FPT at each time point. **p*<0.05 vs baseline; † *p*<0.01 vs baseline; ‡ *p*≤0.001 vs baseline.

Supplementary Table 4: Multiple-imputed data: Mean baseline HR-QoL norm-based and PWI scores, the within-group changes relative to baseline and net between-group differences for the change over the 18-month intervention period in the dual-task functional power training (DT-FPT) and control (CON) groups.

|  | **DT-FPT** | | **CON** | | **Intervention effects** | | |
| --- | --- | --- | --- | --- | --- | --- | --- |
|  | **n** | **Mean ± SD or**  **(95% CI)** | **n** | **Mean ± SD or**  **(95% CI)** | **Estimated group**  **Differences**  **(95% CI)^a^** | ***p*-values**  **Model 1 \| Model 2** | |
| **Physical functioning** | | |  |  |  |  | |
| Baseline | 155 | 40.3 (38.5, 42.0) | 144 | 39.5 (37.6, 41.3) |  |  | |
| ∆ 6 months | 155 | 0.35 (-0.94, 1.65) | 144 | -1.29 (-2.55, -0.04)***** | 1.82 (0.03, 3.62) | **0.046** \| 0.077 |  |
| ∆ 12 months | 155 | 0.75 (-1.13, 2.62) | 144 | -0.57 (-2.05, 0.90) | 1.55 (-0.85, 3.96) | 0.205 \| 0.218 |  |
| ∆ 18 months | 155 | -1.00 (-2.84, 0.83) | 144 | **-1.80 (-3.17, -0.42)*** | 0.98 (-1.20, 3.17) | 0.377 \| 0.442 |  |
| **Role physical** | |  |  |  |  |  |  |
| Baseline | 155 | 45.0 (43.4, 46.6) | 144 | 44.2 (42.5, 45.9) |  |  |  |
| ∆ 6 months | 155 | 0.58 (-0.93, 2.09) | 144 | -1.39 (-3.11, 0.33) | **2.35 (0.10, 4.60)** | **0.041** \| **0.031** |  |
| ∆ 12 months | 155 | -0.75 (-2.38, 0.89) | 144 | -0.14 (-1.92, 1.63) | -0.23 (-2.84, 2.38) | 0.865 \| 0.983 |  |
| ∆ 18 months | 155 | **-2.78 (-4.67, -0.88)**† | 144 | **-1.91 (-3.66, -0.15)*** | -0.53 (-2.94, 1.87) | 0.947 \| 0.839 |  |
| **Bodily pain** |  |  |  |  |  |  |  |
| Baseline | 155 | 44.9 (43.2, 46.7) | 144 | 43.5 (41.5, 45.4) |  |  |  |
| ∆ 6 months | 155 | 0.09 (-1.95, 2.12) | 144 | -0.30 (-2.15, 1.54) | 1.13 (-1.57, 3.83) | 0.410 \| 0.363 |  |
| ∆ 12 months | 155 | -0.91 (-2.98, 1.16) | 144 | 0.43 (-1.47, 2.34) | -0.72 (-3.75, 2.31) | 0.643 \| 0.557 |  |
| ∆ 18 months | 155 | -0.63 (-2.81, 1.55) | 144 | -0.08 (-1.95, 1.78) | 0.10 (-2.81, 3.00) | 0.948 \| 0.880 |  |
| **General health** | |  |  |  |  |  |  |
| Baseline | 155 | 47.6 (46.3, 48.8) | 144 | 46.7 (45.2, 48.2) |  |  |  |
| ∆ 6 months | 155 | 0.68 (-0.32, 1.69) | 144 | 0.88 (-0.48, 2.24) | -0.002 (-1.59, 1.58) | 0.998 \| 0.658 |  |
| ∆ 12 months | 155 | 0.37 (-1.03, 1.78) | 144 | 0.64 (-0.70, 1.97) | 0.004 (-1.85, 1.86) | 0.997 \| 0.666 |  |
| ∆ 18 months | 155 | -0.24 (-1.80, 1.31) | 144 | -0.03 (-1.56, 1.50) | -0.06 (-2.05, 1.93) | 0.952 \| 0.790 |  |
| **Vitality** |  |  |  |  |  |  |  |
| Baseline | 155 | 48.8 (47.3, 50.3) | 144 | 49.0 (47.4, 50.6) |  |  |  |
| ∆ 6 months | 155 | -0.003 (-1.63, 1.62) | 144 | 0.18 (-1.45, 1.80) | -0.25 (-2.33, 1.83) | 0.816 \| 0.889 |  |
| ∆ 12 months | 155 | -0.52 (-1.86, 0.82) | 144 | -0.69 (-2.25, 0.86) | 0.35 (-1.89, 2.16) | 0.896 \| 0.732 |  |
| ∆ 18 months | 155 | -1.36 (-3.23, 0.50) | 144 | 0.08 (-1.27, 1.42) | -1.53 (-3.68, 0.62) | 0.162 \| 0.169 |  |
| **Social functioning** | | |  |  |  |  |  |
| Baseline | 155 | 49.2 (47.7, 50.7) | 144 | 49.3 (47.8, 50.9) |  |  |  |
| ∆ 6 months | 155 | -0.25 (-1.80, 1.29) | 144 | **-1.91 (-3.68, -0.14)*** | 1.57 (-0.82, 3.96) | 0.197 \| 0.247 |  |
| ∆ 12 months | 155 | -0.73 (-2.63, 1.18) | 144 | -0.55 (-1.65, 0.54) | -0.24 (-2.27, 1.80) | 0.819 \| 0.774 |  |
| ∆ 18 months | 155 | **-3.29 (-5.77, -0.82)**† | 144 | **-1.84 (-3.49, -0.19)*** | -1.54 (-4.44, 1.35) | 0.296 \| 0.257 |  |
| **Role emotional** | |  |  |  |  |  |  |
| Baseline | 155 | 46.4 (44.5, 48.3) | 144 | 46.9 (45.0, 48.8) |  |  |  |
| ∆ 6 months | 155 | 1.86 (-0.13, 3.86) | 144 | -1.30 (-3.34, 0.75) | 2.88 (0.24, 5.52) | **0.033** \| 0.055 |  |
| ∆ 12 months | 155 | -0.70 (-2.82, 1.42) | 144 | -1.51 (-3.61, 0.58) | 0.60 (-2.41, 3.62) | 0.694 \| 0.594 |  |
| ∆ 18 months | 155 | -1.69 (-4.61, 1.23) | 144 | **-3.28 (-5.73, -0.82)**† | 1.29 (-1.98, 4.55) | 0.439 \| 0.579 |  |
| **Mental health** | |  |  |  |  |  |  |
| Baseline | 155 | 49.6 (48.1, 51.0) | 144 | 50.0 (48.5, 51.5) |  |  |  |
| ∆ 6 months | 155 | 0.09 (-1.47, 1.66) | 144 | -0.0002 (-1.50, 1.50) | -0.15 (-1.88, 1.59) | 0.868 \| 0.719 |  |
| ∆ 12 months | 155 | 0.40 (-1.18, 1.98) | 144 | 0.52 (-1.08, 2.12) | -0.37 (-2.37, 1.63) | 0.716 \| 0.556 |  |
| ∆ 18 months | 155 | -0.79 (-2.92, 1.39) | 144 | -0.45 (-1.91, 1.00) | -0.60 (-2.97, 1.77) | 0.620 \| 0.381 |  |
| **Physical Component Summary** | | |  |  |  |  |  |
| Baseline | 155 | 43.0 (41.4, 44.6) | 144 | 41.6 (39.6, 43.2) |  |  |  |
| ∆ 6 months | 155 | 0.14 (-0.95, 1.24) | 144 | -0.63 (-1.82, 0.56) | 1.18 (-0.47, 2.83) | 0.161 \| 0.106 |  |
| ∆ 12 months | 155 | -0.16 (-1.68, 1.37) | 144 | 0.09 (-1.12, 1.30) | 0.20 (-1.96, 2.36) | 0.854 \| 0.773 |  |
| ∆ 18 months | 155 | -1.27 (-2.86, 0.32) | 144 | -0.70 (-1.86, 0.46) | -0.20 (-2.17, 1.77) | 0.845 \| 0.897 |  |
| **Mental Component Summary** | | |  |  |  |  |  |
| Baseline | 155 | 51.0 (49.5, 52.4) | 144 | 51.9 (50.3, 53.4) |  |  |  |
| ∆ 6 months | 155 | 0.66 (-0.83, 2.14) | 144 | -0.58 (-2.01, 0.85) | 0.75 (-0.87, 2.38) | 0.364 \| 0.447 |  |
| ∆ 12 months | 155 | -0.37 (-2.13, 1.40) | 144 | -0.60 (-2.07, 0.88) | -0.20 (-2.37, 1.96) | 0.853 \| 0.872 |  |
| ∆ 18 months | 155 | -1.63 (-3.89, 0.63) | 144 | -1.55 (-3.23, 0.12) | -0.60 (-3.06, 1.86) | 0.632 \| 0.439 |  |
| **Global HR-QoL** | |  |  |  |  |  |  |
| Baseline | 155 | 46.5 (45.3, 47.7) | 144 | 46.1 (44.9, 47.4) |  |  |  |
| ∆ 6 months | 155 | 0.43 (-0.463, 1.31) | 144 | -0.65 (-1.76, 0.45) | 1.19 (-0.13, 2.50) | 0.076 \| 0.071 |  |
| ∆ 12 months | 155 | -0.27 (-1.12, 0.58) | 144 | -0.24 (-1.06, 0.58) | 0.05 (-1.20, 1.30) | 0.937 \| 0.812 |  |
| ∆ 18 months | 155 | **-1.46 (-2.81, -0.12)*** | 144 | **-1.16 (-1.93, -0.40)**† | -0.23 (-1.64, 1.19) | 0.755 \| 0.731 |  |
| **Satisfaction with life** | | |  |  |  |  |  |
| Baseline | 155 | 78.4 (75.8, 81.1) | 144 | 78.9 (76.0, 81.9) |  |  |  |
| ∆ 6 months | 155 | 0.66 (-2.16, 3.47) | 144 | 1.43 (-0.52, 3.38) | -1.01 (-4.25, 2.22) | 0.538 \| 0.470 |  |
| ∆ 12 months | 155 | -0.52 (-3.14, 2.10) | 144 | 2.30 (-0.47, 5.07) | -3.04 (-7.19, 1.12) | 0.151 \| 0.116 |  |
| ∆ 18 months | 155 | 0.99 (-2.84, 4.26) | 144 | 0.41 (-2.67, 3.48) | 0.35 (-3.80, 4.50) | 0.869 \| 0.913 |  |
| **Personal Well-being Index Composite** | | |  |  |  |  |  |
| Baseline | 155 | 80.3 (78.4, 82.2) | 144 | 79.3 (77.0, 81.6) |  |  |  |
| ∆ 6 months | 155 | 1.07 (-0.77, 2.91) | 144 | 1.38 (-0.04, 2.81) | 0.09 (-2.22, 2.40) | 0.805 \| 0.827 |  |
| ∆ 12 months | 155 | 0.07 (-1.93, 2.07) | 144 | **2.72 (0.71, 4.72)**† | -2.29 (-5.25, 0.68) | 0.131 \| 0.082 |  |
| ∆ 18 months | 155 | 0.31 (-1.79, 2.40) | 144 | 1.30 (-0.63, 3.24) | -0.65 (-3.49, 2.18) | 0.651 \| 0.533 |  |

*Note.* Baseline values are reported as means ± SD. For HR‑QoL and life satisfaction outcomes, higher scores indicate better outcomes. For change scores, positive values reflect improvement while negative values reflect decline. Within-group and estimated between-group differences are presented as means with 95% CI, adjusted for clustering. P-values for group differences were derived from linear mixed models with random intercepts for villages: Model 1 (adjusted for baseline values and clustering) and Model 2 (adjusted for age, sex, education, cardiometabolic status, baseline values, and clustering). HR-QoL: Health-related quality of life. Personal Wellbeing Index composite is the average of PWI subdomains. Satisfaction with Life was a single-item measure multiplied by 10. Bolded values indicate statistically significant within-group changes relative to baseline after adjusting for clustering, and statistically significant estimated between-group differences. ^a^ Estimated between-group differences (95% CIs) represent coefficients from Model 1, rather than by subtracting within-group changes from baseline for CON from within-group changes for DT-FPT at each time point. **p*<0.05 vs baseline. † *p*<0.01 vs baseline.

Supplementary Table 5: Mean baseline Depression, Anxiety and Stress Scale values, the within-group changes relative to baseline and net between-group differences for the change over the 18-month intervention period in the dual-task functional power training (DT-FPT) and control (CON) groups.

|  | **DT-FPT** | | | **CON** | | **Intervention effects** | | |
| --- | --- | --- | --- | --- | --- | --- | --- | --- |
|  | **n** | **Mean ± SD or**  **(95% CI)** | | **n** | **Mean ± SD or**  **(95% CI)** | **Estimated group**  **Differences**  **(95% CI)^a^** | ***p*-values**  **Model 1 \| Model 2** | |
| **DASS-21: Depression** | | | |  |  |  |  | |
| Baseline | 154 | | 3.44 ± 4.83 | 143 | 4.0 ± 5.85 |  |  | |
| ∆ 6 months | 118 | | 0.04 (-0.76, 0.85) | 118 | -0.22 (-0.86, 0.42) | 0.17 (-0.80, 1.14) | 0.727 \| 0.573 |  |
| ∆ 12 months | 113 | | 0.80 (-0.03, 1.62) | 114 | -0.05 (-1.02, 0.92) | 0.80 (-0.52, 2.12) | 0.235 \| 0.137 |  |
| ∆ 18 months | 113 | | 0.53 (-0.65, 1.72) | 114 | 0.11 (-0.77, 0.99) | 0.41 (-1.00, 1.82) | 0.572 \| 0.354 |  |
| **DASS-21: Anxiety** | | | |  |  |  |  |  |
| Baseline | 154 | | 3.38 ± 4.62 | 143 | 4.22 ± 5.36 |  |  |  |
| ∆ 6 months | 118 | | -0.33 (-1.15, 0.50) | 118 | **-1.00 (-1.77, -0.23)*** | 0.26 (-0.59, 1.10) | 0.552 \| 0.414 |  |
| ∆ 12 months | 113 | | **0.83 (0.07, 1.60)*** | 114 | **-1.28 (-2.00, -0.56)**‡ | **1.83 (0.72, 2.95)** | **0.001 \| 0.001** |  |
| ∆ 18 months | 113 | | -0.12 (-0.87, 0.63) | 114 | **-1.04 (-1.65, -0.42)**‡ | **0.69 (-0.19, 1.58)** | 0.125 \| **0.045** |  |
| **DASS-21: Stress** | | | |  |  |  |  |  |
| Baseline | 154 | | 5.45 ± 6.05 | 143 | 5.37 ± 5.98 |  |  |  |
| ∆ 6 months | 118 | | 0.47 (-0.42, 1.36) | 118 | -0.22 (-0.98, 0.53) | 0.84 (-0.15, 1.83) | 0.095 \| 0.076 |  |
| ∆ 12 months | 113 | | 0.60 (-0.63, 1.82) | 114 | -0.63 (-1.70, 0.44) | 1.31 (0.10, -2.71) | 0.069 \| 0.061 |  |
| ∆ 18 months | 113 | | -0.24 (-1.47, 0.99) | 114 | 0.05 (-0.73, 0.83) | -0.17 (-1.40, 1.07) | 0.791 \| 0.949 |  |

*Note.* Baseline values are reported as means ± SD. For DASS-21 outcomes, lower scores reflect reduced psychological distress. Negative change scores indicate improvement in depression, anxiety, and stress symptoms, whereas positive change scores indicate increased symptom severity. Within-group and estimated between-group differences are presented as means with 95% CI, adjusted for clustering. P-values for group differences were derived from linear mixed models with random intercepts for villages: Model 1 (adjusted for baseline values and clustering) and Model 2 (adjusted for age, sex, education, cardiometabolic status, baseline values, and clustering). At baseline, DASS-21 questionnaires were not completed by two participants (DT-FPT: n=1; CON: n=1). ^a^ Estimated between-group differences (95% CIs) were calculated from coefficients from Model 1, rather than by subtracting within-group changes from baseline for CON from within-group changes for DT-FPT at each time point. Bolded values indicate statistically significant within-group changes relative to baseline after adjusting for clustering, and statistically significant estimated between-group differences. **p*<0.05 vs baseline; ‡ *p*≤0.001 vs baseline.
